# Potential Climate Impact Variations Due to Fueling Behavior of Plug-in Hybrid Vehicle Owners in the US

**DOI:** 10.1021/acs.est.0c03796

**Published:** 2020-12-16

**Authors:** Paul Wolfram, Edgar G. Hertwich

**Affiliations:** † Center for Industrial Ecology, School of the Environment, Yale University, 195 Prospect Street, New Haven, Connecticut 06511, United States; ‡ Industrial Ecology Programme, Department of Energy and Process Engineering, Norwegian University of Science and Technology, 7491 Trondheim, Norway

## Abstract

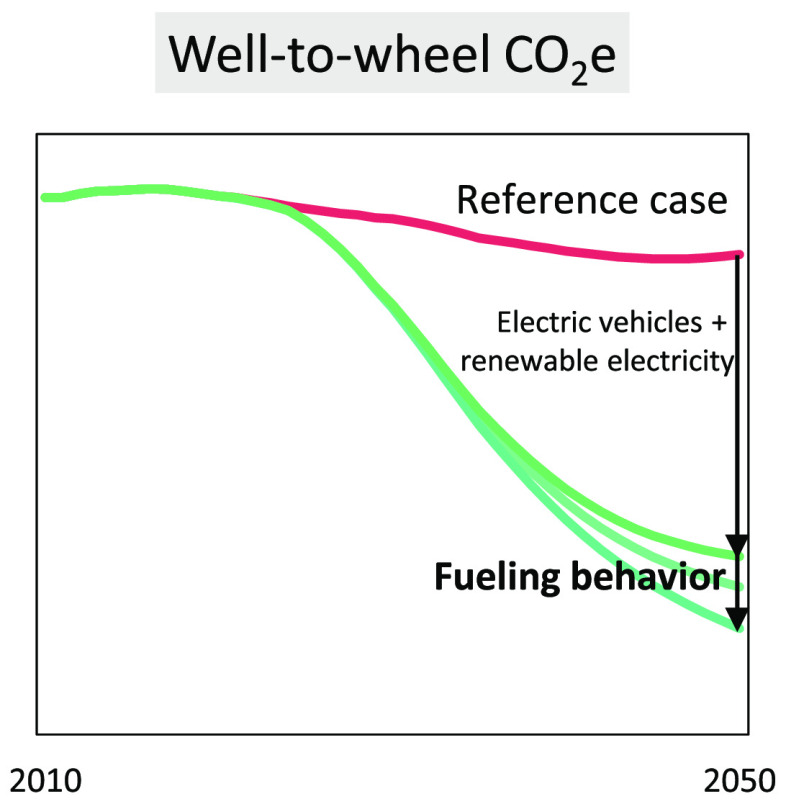

With the expected rapid growth of renewable electricity generation, charging plug-in hybrid electric vehicles (PHEVs) from the grid promise ever higher reductions in CO_2_ emissions. Previous analyses have found that the share that PHEVs are driven in electric mode can differ substantially depending on region, battery size, and trip purpose. Here, we provide a first fleet-wide emissions mitigation potential of US-based PHEV drivers adopting high or low shares of electric driving. Specifically, we illustrate scenarios of different combinations of PHEV uptake, renewable electricity generation shares, and PHEV fueling behavior. Across 21 analyzed scenarios, annual greenhouse gas (GHG) emissions of the light-duty vehicle (LDV) fleet could differ by an average of 21% (5–43% range) in 2050 depending alone on the fueling behavior of PHEV drivers. This behavior could further determine the discharge of about 1.3 (0.7–1.9) Gt CO_2_ (or roughly one year of current emissions) over the next three decades, significantly influencing the feasibility of reaching an 80% emission reduction target for the LDV sector. Governments can nudge PHEV drivers toward environmentally favorable fueling behavior. We discuss several options for nudging, including charging infrastructure availability, battery design, and consumer education.

## 1 Introduction

Since a record high in 2007, GHG emissions from the US electricity sector have fallen by almost a third in subsequent years.^1^ This positive development has been contrasted by steadily growing GHG emissions from the transport sector in the same period,^1^ despite substantial efficiency gains.^2^ Thus, one promising measure to reduce transport GHG emissions, especially in the LDV sector, is to increasingly electrify transport,^3^ capitalizing on the falling carbon intensity of electricity.

However, only a few of today’s commercially available battery electric vehicle (BEV) models offer a sufficient range for long-distance trips on a single charge. With 515 and 595 km (320 and 370 miles),^4^ only Tesla’s Model 3 and Model S long-range variants offer a driving range comparable to the 650–800 km (400–500 miles) that popular conventional cars provide today. Due to their significant price tag, both models may be reserved to a small percentage of the population however. A lack of charging infrastructure, load shedding, and high cost of electricity can pose additional challenges, especially for developing countries, making it difficult to transition to fully electric cars.^5^

This is where PHEVs have an important advantage over BEVs: PHEVs can run on both electricity and liquid fuel. Once the battery is depleted, the driver can continue driving on gasoline. Even though the electric range of most of today’s PHEVs is limited to below 48 km (30 miles),^6^ one study finds that long-range PHEVs electrify as many annual miles driven as BEVs.^7^ Another advantage is that the smaller battery of a PHEV leads to a smaller price increment over conventional cars. Thus, PHEVs could play a significant role in the future according to IEA projections.^8^

For the most effective mitigation of GHG emissions, it is, however, crucial that PHEVs are mostly driven on electricity and in areas with a suitably clean supply. Much analysis has therefore been devoted to the share of electric driving of individual PHEVs. For example, using data from the National Household Travel Survey, MacPherson et al. estimate that PHEVs are driven on electricity 60.2% of the time on average in the US, slightly lower than the estimate of 63.5% by the US Environmental Protection Agency (EPA).^9^ This fraction is usually termed the utility factor (UF) of a PHEV.^10^ MacPherson et al. also find that the regional heterogeneity of the UF ranges from below 0.6 in the Midwest and the Northeast to above 0.8 in Alaska (estimated from Figure 7 therein).^9^ Ligterink and Eijk find an average UF of 0.33 in The Netherlands.^11^ Including business travelers who hardly charge lowers the UF further to 0.24. Goebel and Plötz analyze data of 1,768 Chevrolet Volt’s on-board diagnostics systems and find UFs ranging from 0.14 to 1.00, with a median of 0.80 and a mean of 0.77.^12^ Similarly, Raghavan and Tal find UFs up to just under 1.0 for Ford C-MAX (32-km/20-mile electric range) and Chevrolet Volt (56–85 km/35–53-mile electric range) users, while Toyota Prius (18-km/11-mile electric range) drivers do not reach UFs higher than 50%.^13^ The authors also observe that actual “real-world” UFs are somewhere between 60 and 103% of EPA’s estimates, which are based on drive-cycle simulations, indicating that EPA figures overestimate the UF of certain PHEV models.

Researchers observe a similar fueling behavior of other bifuel vehicle drivers. During a 10-year trial, Johns et al. find that alternative fuels, such as E-85 (a blend containing up to 85% ethanol and 15% gasoline by volume), compressed natural gas, or liquefied petroleum gas, accounted for only 30% of fuels used in bifuel vehicles, meaning that the majority of miles were fueled by gasoline.^14^ They further find that convenience, informal communication with peers, as well as incentives and sanctions have a big effect on alternative fuel use.

Other important factors include (1) the timing of charging, (2) ride-sourcing, and (3) ambient temperature. Regarding (1), Axsen et al. report that UFs are reduced if only off-peak charging is available compared to when charging is available at all times.^15,16^ Despite the lower UF, GHG emissions from combustion and upstream processes can be reduced when times of peak electricity generation, which often are dominated by more carbon-intensive energy sources, can be avoided. (2) The International Transport Forum estimates that the UF of ride-sourced PHEVs is almost half that of a private PHEV largely due to “deadheading”, i.e., empty trips of ride-sourced vehicles to passenger pick-up locations.^17^ (3) Finally, Wu et al. show that extreme ambient temperatures can significantly reduce the fuel economy of PHEVs and other powertrains.^18^ Higher energy consumption reduces the electric range of PHEVs which can lead to reduced UFs. Climate change is likely to lead to more heat extremes in the future^19^ which could further exacerbate this issue.

Systemic models, such as transport sector models, energy systems models, or integrated assessment models, are used to explore potential sectoral or holistic pathways of climate change mitigation. While these models increasingly improve their approximation of consumer behavior,^20,21^ fueling and charging behaviors are often modeled in a rather simplistic fashion.^22^ For example, Karplus et al. define UFs exogenously in the EPPA integrated assessment model.^23^

To conclude, the fuel use behavior of PHEV drivers varies substantially, depending on trip purpose (e.g., business vs nonbusiness trips), climate, electricity prices, and availability. These extremes could be even more pronounced in the future, depending on the development of charging infrastructure, climate impacts on PHEV battery performance, and user education. While the fueling behavior (and sometimes the corresponding variation in climate impact) has been studied quite extensively for individual PHEVs or smaller fleets of government-operated bifuel vehicles, the authors are unaware of any study attempting to compute a fleet-wide estimate of well-to-wheel (WTW) GHG emissions at the national level using a detailed transport scenario model. As such, we regard this to be the first study to analyze scenarios of climate impacts of the fueling behavior of US PHEV drivers considering various dynamics of the energy system.

## 2 Methods and Data

### 2.1 Model Overview

For our analysis, we use the LAVE-Trans (Light-duty Alternative Vehicle Energy Transitions) model.^24−28^ LAVE-Trans is a transportation scenario model forecasting WTW GHG emissions from US LDVs. WTW emissions include emissions of the entire energy chain from the production of energy carriers to their final use. Estimates are provided annually for the time period 2005 to 2050.

### 2.2 Vehicle Choice

Technology choice is endogenized in LAVE-Trans through a nested discrete choice model in which six powertrain technologies are available: internal combustion engine vehicles (ICEVs), hybrid electric vehicles (HEVs), PHEVs, BEVs, compressed natural gas vehicles, and hydrogen fuel cell electric vehicles. Consumers can further choose between two vehicle classes: passenger cars and light trucks. For each powertrain and segment, the model distinguishes several characteristics such as vehicle purchase price, fuel economy/fuel costs, driving range, charging and fuel station availability, model diversity, and maintenance cost. The model also accounts for available vehicle subsidies which are subtracted from vehicle purchase prices and provides an option to model a carbon tax on energy carriers, increasing their price as a function of their carbon content and the magnitude of the tax. While fuel economy and CO_2_ standards cannot be fully modeled, LAVE-Trans assumes by default that the costs of ICEVs are going to increase in the future which can be primarily seen as an effect of increasingly stringent regulations.

The model, however, considers different consumer risk groups, i.e., innovators and early adopters (16%), early majority (34%), late majority (34%), and laggards (16%). These groups are distinguished by their willingness to pay for above-mentioned vehicle characteristics. Each consumer picks the vehicle which provides them with the lowest disutility/cost. The probability distribution of different consumers picking certain vehicles is directly translated into vehicle sales shares in a given year. A vintage-based vehicle fleet stock module then translates the inflows of new vehicles and the outflows of retired vehicles into the current vehicle stock and the corresponding total travel demand for each year.

### 2.3 Vehicle Types

**Figure 1:**
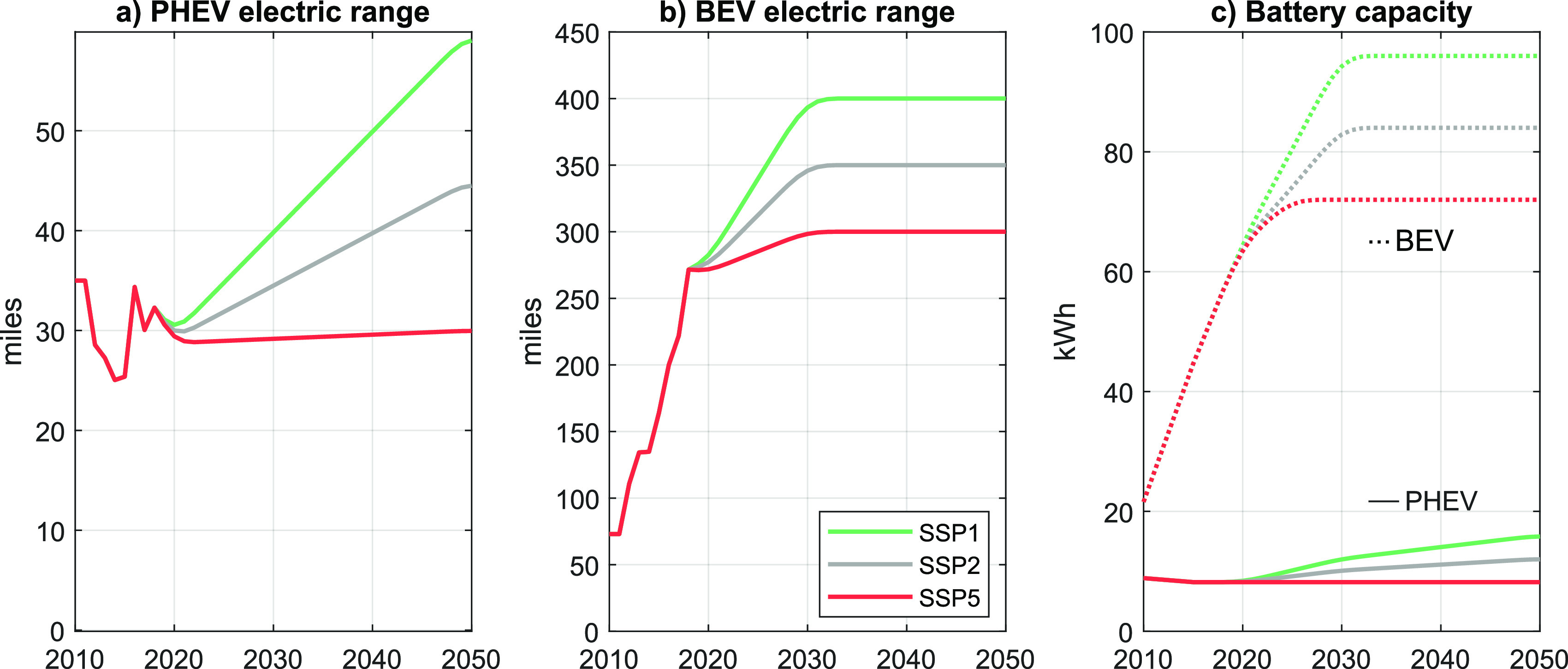
Estimated sales-weighted electric driving range and battery capacity of BEV and PHEV. SSP = shared socioeconomic pathway; BEV = battery electric vehicle; PHEV = plug-in hybrid electric vehicle.

Current PHEVs are modeled to have a 48-km (30-mile) all electric range (Figure 1a), which corresponds well to the sales-weighted average electric range of the nine highest sold PHEVs, each with cumulative sales above 15,000 units between 2011 and 2019 (estimated using data on the electric range by model from the EPA’s fuel economy database^4^ and vehicle sales data from the Alternative Fuels Data Center^29^). These nine models represent 81% of cumulative PHEV sales in that time period. In SSP5, we assume that the PHEV electric range remains constant over the studied time frame, while increasing ranges are presumed in SSP2 and SSP1 (see section S1.1).

Similarly, in SSP1, we assume that the driving range of BEVs increases up to 644 km (400 miles) by around 2030 (Figure 1b), which is at the lower end of the range that current gasoline cars provide. The sales-weighted average range of BEVs already increased from 121 km (75 miles) in 2011 to around 435 km (270 miles) in 2019. Figure 1c illustrates the corresponding battery capacities, assuming a charging loss factor of 5%. Detailed cost estimates for all powertrains through 2030 are largely based on Wolfram and Lutsey.^30^ Here we extend the time horizon to 2050 and update BEV and PHEV battery cost estimates in line with Lutsey and Nicholas,^31^ BNEF,^32^ Edelenbosch et al.,^33^ and Ziegler and Trancik^34^ (see sections 2.5 and S1.3 for more details).

### 2.4 Emission Factors

**Figure 2:**
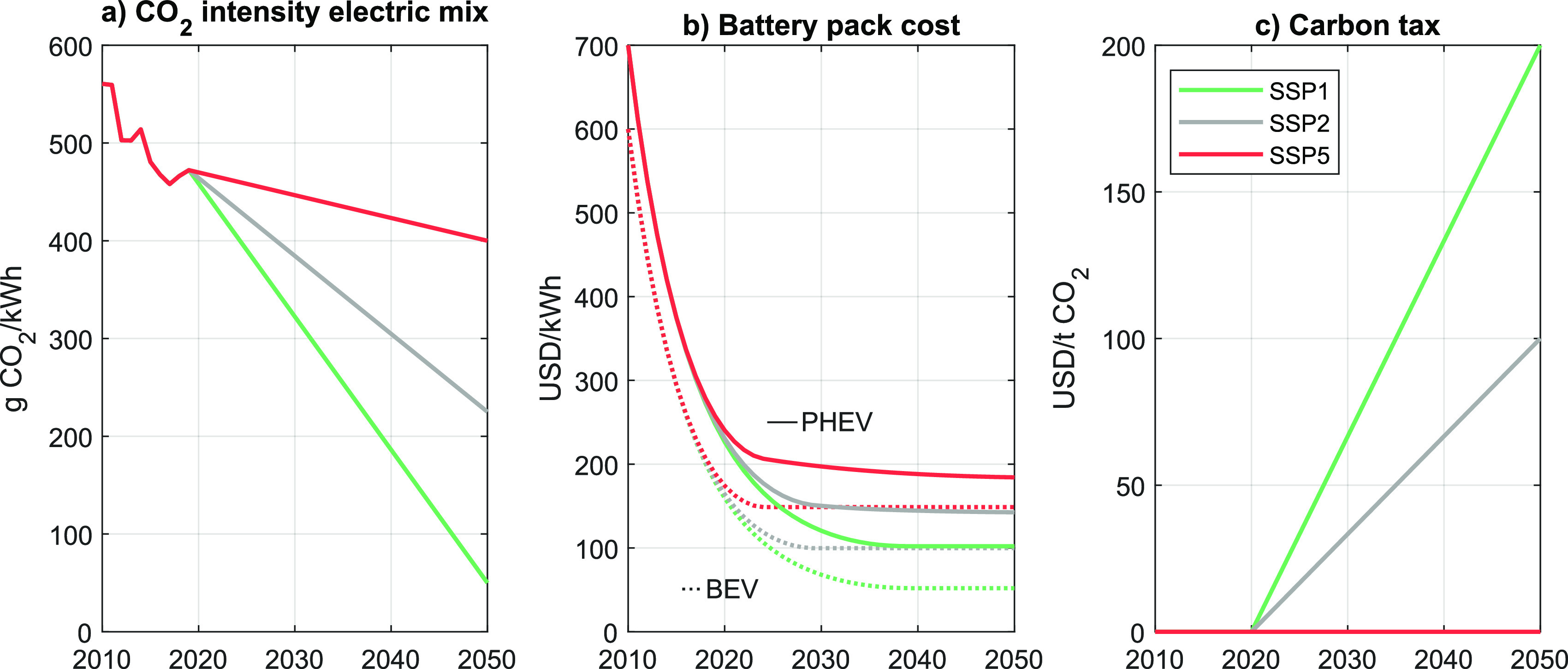
Select scenario input parameters. SSP = shared socioeconomic pathway; PHEV = plug-in hybrid electric vehicle; BEV = battery electric vehicle.

LAVE-Trans provides detailed emission factors for several energy carriers on a WTW basis, including gasoline, corn ethanol, cellulosic ethanol, biofuel from cellulosic pyrolysis, liquefied coal, liquefied gas, electricity, hydrogen, compressed natural gas, and liquified petroleum gas. Gasoline can be blended with several of these fuels. In this work, we adopt the standard assumptions of the LAVE-Trans model on gasoline according to which conventional gasoline is increasingly blended with other drop-in fuels. Specifically, the share of conventional gasoline falls from 94% in 2010 to 74% in 2050, while the shares of corn ethanol, cellulosic ethanol, and liquefied coal increase accordingly. As a result, the carbon intensity of gasoline falls slightly from 318 g CO_2_e/kWh in 2010 to 297 g CO_2_e/kWh in 2050. Also the carbon intensity of the electricity mix falls over time, which is in line with recent developments.^1^ Depending on the scenario, the decarbonization of the electricity grid differs in magnitude however (see next subsection and Figure 2a). Our model assumes average, not marginal emissions factors of electricity generation.

### 2.5 Scenarios of the Energy System

We develop three main scenarios following the shared socioeconomic pathway (SSP) framework.^35^ From the five available SSPs, we adopt SSP1, SSP2, and SSP5. These three scenarios differ in terms of challenges to GHG mitigation. SSP1 (“sustainability”) faces the lowest mitigation challenges, whereas SSP2 (“middle of the road”) faces medium challenges, and SSP5 (“fossil-fueled development”) faces high mitigation challenges. We do not adopt SSP3 or SSP4 for one major reason. The main difference between SSP3 and SSP5 is the differing degree of climate change adaption challenges, while mitigation challenges are assumed to be similar (see Figure 1 in ref (35)). The same relationship exists for SSP2 and SSP4. Since we do not model climate change adaption measures in this work, the pairs SSP2/SSP4 and SSP3/SSP5 can be regarded as equivalent and would not yield different modeling outcomes.

#### 2.5.1 Electricity Carbon Intensity

In SSP1, we assume that the carbon intensity of the electricity mix falls from 561 g CO_2_e/kWh in 2010 down to 50 g CO_2_e/kWh in 2050 due to significant uptake of renewable electricity as well as CO_2_ capture and efficiency improvements of remaining fossil-fueled power plants. SSP5 is characterized by a moderate reduction down to 400 g CO_2_e/kWh in the same time period. As the name suggests, SSP2 follows the in-between path and reaches 225 g CO_2_e/kWh (Figure 2a).

#### 2.5.2 Battery Costs

The costs of battery packs are assumed to decrease rapidly in SSP1. BEV batteries reach a floor price of 50 USD/kWh by 2050, while PHEV battery costs fall down to 100 USD/kWh (Figure 2b). PHEV batteries are assumed to decrease at a slower rate, because they have a smaller storage capacity yet need to provide high power.^24,30^ However, the incremental cost of PHEV batteries over BEV batteries also falls over time. These assumptions are comparable to those made in recent publications. For example, Lutsey and Nicholas assume that BEV battery packs could reach 64–73 USD/kWh by 2030, while PHEV battery packs arrive at 86–88 USD/kWh.^31^ Berckmans et al. calculate that BEV pack costs could fall down to 50–80 USD/kWh by 2030.^36^ Estimating from Figure 1 in Edelenbosch et al., the authors assume pack costs of about 65 USD/kWh by 2030 and 50 USD/kWh by 2050 in their most optimistic scenario.^33^ A recent consulting report estimates a pack price of 73 USD/kWh by 2030.^32^ The two latter sources do not mention differences in relative (USD/kWh) costs between BEV and PHEV battery cost. Also note that first industry claims of battery costs as low as 80 USD/kWh have been made as of 2020.^37^ More pessimistic cost trajectories^30,33^ for BEV batteries roughly translate to 150 USD/kWh by 2050, which we assume representative for SSP5. Accordingly, the middle-of-the road scenario assumes 100 USD/kWh (for more details see section S1.3).

#### 2.5.3 Carbon Tax

In SSP1, we further assume that a carbon tax is introduced in 2021, which linearly ramps up to 200 USD/ton CO_2_ by midcentury. While no carbon tax is assumed in SSP5, SSP2 again follows the in-between path (Figure 2c). Consequently, prices of carbon-intensive energy carriers significantly increase in SSP1. For example, gasoline reaches 1.52 USD/L (5.75 USD/gal) by 2050. Without the added cost of a carbon tax, the gasoline price moderately increases to about 1.04 USD/L (3.93 USD/gal) in SSP5 which is somewhat higher than the assumed 0.90 USD/L (3.40 USD/gal) in the reference scenario of the 2020 Annual Energy Outlook.^38^ Electricity prices develop in similar ways in all three scenarios but react to different underlying mechanisms. In SSP1, the electricity price is less affected by the carbon tax due to strong reductions in carbon intensity of the electric grid (Figure 2a). However, the added cost of the massive expansion of renewable electricity still causes consumer electricity prices to increase from about 11 ct./kWh to about 16 ct./kWh by 2050. Due to the impact of the carbon tax, SSP2 and SSP5 experience similar price increases up to 18 and 16 ct./kWh by 2050.

#### 2.5.4 Fueling Behavior

For each SSP, we first illustrate a case in which UFs grow in accordance with larger battery capacities, that is up to 0.9 in SSP1 and up to 0.75 in SSP2, while battery capacities and UFs remain constant in SSP5. We then illustrate cases in which UFs remain below these upper estimates despite growing battery capacities. Reasons therefore could be increased demand for ride-sourcing services, inadequate recharging infrastructure, and potential restrictions for PHEV users to use all charging bays (for more details and some back-of-the-envelope calculations refer to section S1.2). We conservatively estimate that these factors could reduce fleet-wide average UFs by about 20–25%.

## 3 Results

### 3.1 Vehicle Market Shares

**Figure 3:**
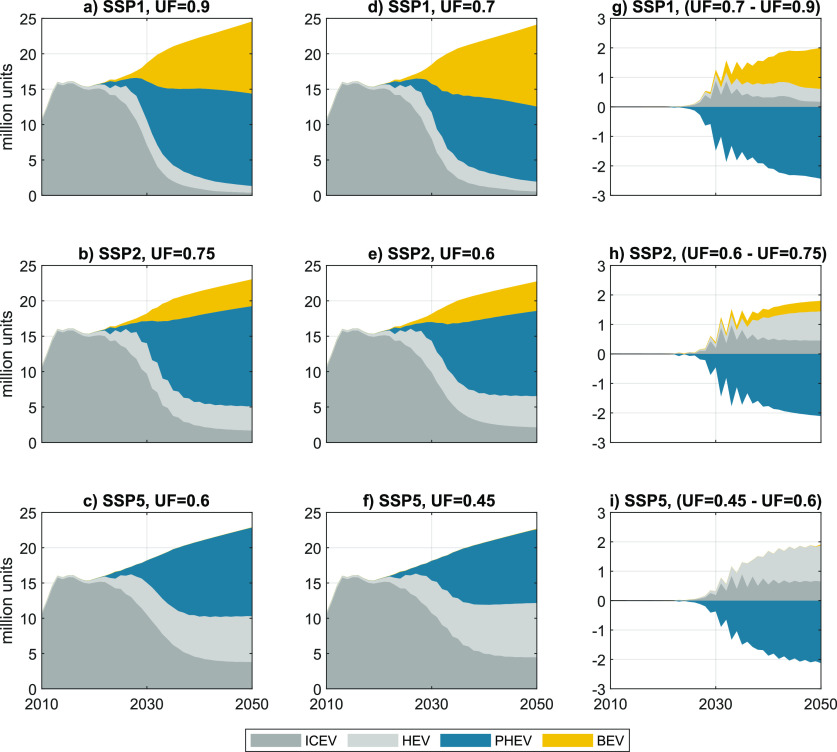
Vehicle sales under different SSPs with high UFs (a–c) and with low UFs (d–f) and differences in sales due to variations in UFs (g–i). SSP = shared socioeconomic pathway; ICEV = internal combustion engine vehicle; HEV = hybrid electric vehicle; PHEV = plug-in hybrid electric vehicle; BEV = battery electric vehicle.

Sales shares differ substantially in all three scenarios due to the different prevalent conditions described above. BEVs become fully cost-competitive in SSP1 and share the vast majority of the market with PHEVs (Figure 3a). BEVs also attain considerable shares in SSP2, but PHEVs are the dominating drive technology (Figure 3b). SSP5 sees a major uptake of HEVs accompanied by a moderate increase of PHEV sales (Figure 3c). Accordingly, cumulative sales of PHEVs reach 254 million units in SSP1, 242 million units in SSP2, and 210 million units in SSP5. Lower UFs lead to reduced PHEV sales in all scenarios (Figures 3d–f) as increased use of gasoline also raises total cost of ownership. The differences in vehicles sales between the cases with high and low UFs can be seen in detail in Figures 3g–i. The influence of these sales figures on the total vehicle stock and on fleet-wide energy demand is shown in section S2.

### 3.2 Fleet-Wide WTW GHG Emissions

**Figure 4:**
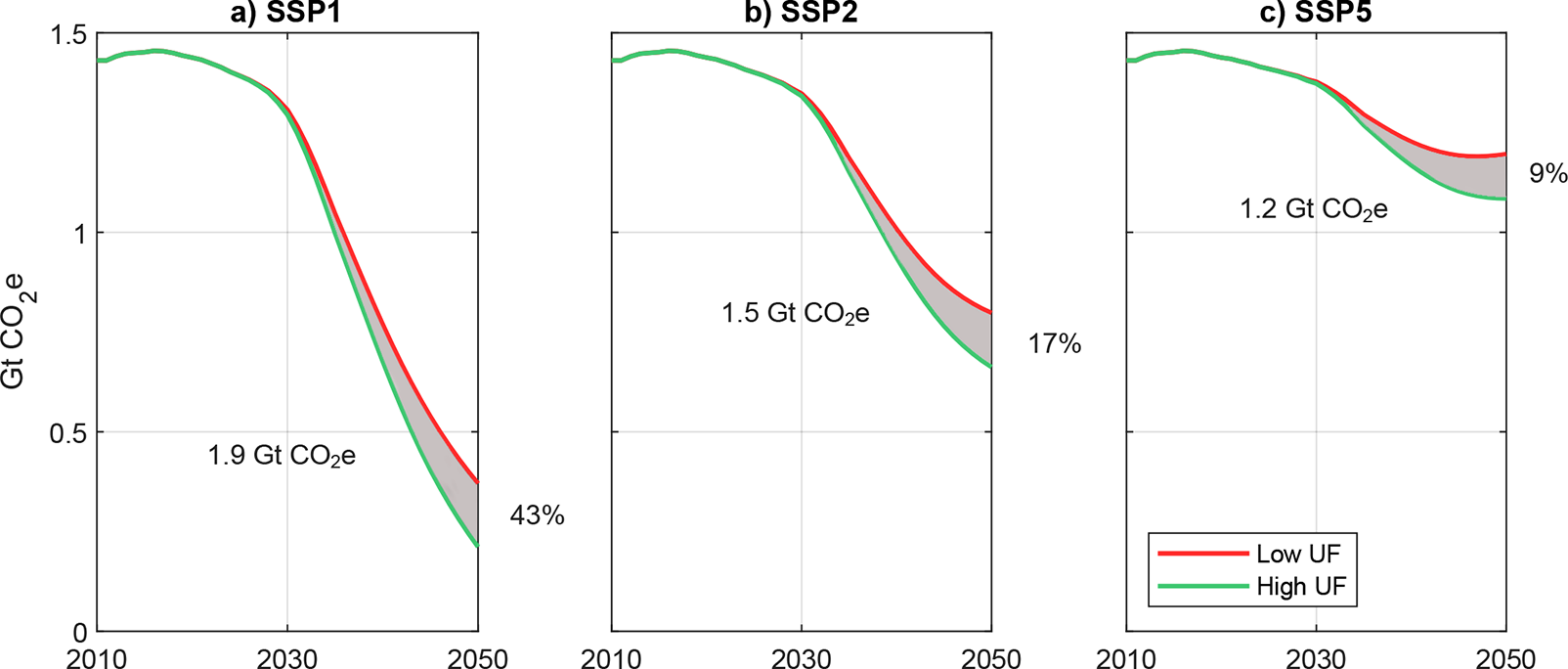
Well-to-wheel greenhouse gas emissions under the three SSPs with varying fueling behavior of plug-in hybrid vehicle drivers. SSP = shared socioeconomic pathway; UF = utility factor.

Fleet-wide WTW GHG emissions are estimated at 1.38 Gt CO_2_e in 2005 and follow a slight upward trend until about 2016, peaking at about 1.45 Gt, after which emissions are beginning to fall in all scenarios albeit at different pace. Assuming increasingly favorable PHEV fueling behavior (UF = 0.9), emissions fall quickly in SSP1, due to the sharp increase of BEVs and PHEVs which are mainly fueled by low-carbon electricity. As a result, GHG emissions reach 0.21 Gt by 2050 (green line in Figure 4a), which is 85% below 2005 levels. Cumulative emissions over the 2005–2050 period sum up to 43.3 Gt CO_2_e. Although SSP5 (UF = 0.6) is dominated by fossil fuels and relatively less efficient vehicles, a moderate reduction in emissions by 21% by 2050 relative to 2005 can be achieved, while cumulative emissions reach 53.6 Gt CO_2_e. This is mainly due to the fact that HEVs are strongly penetrating the market. While HEVs, just like ICEVs, operate on gasoline, their ability to capture braking energy makes them significantly more economical compared to ICEVs. Meanwhile, further reductions are realized due to moderate sales of PHEVs as well as significant efficiency improvements of ICEVs (see section S2.2 for more details). Nestled in between SSP1 and SSP5, SSP2 (UF = 0.75) achieves a 52% reduction in emissions in the analyzed period, mainly due to a stronger uptake of PHEVs and simultaneous reductions in HEV and ICEV sales compared to SSP5. Cumulative emissions arrive at 45.8 Gt CO_2_e.

### 3.3 The Influence of PHEV Fueling Behavior

In the scenarios considered, fueling behavior of PHEV drivers can have a surprisingly high impact on fleet-wide emissions. For example, in SSP1, an increasingly less favorable fueling behavior of PHEV users (UF = 0.7) can lead to missed emission reduction opportunities in the range of 1.9 Gt CO_2_e (compare the gray area between the green and the red line in Figure 4a). With 1.2 and 1.5 Gt CO_2_e, this difference is similar in SSP5 and SSP2 (see gray-shaded areas in Figures 4b and 4c). The potential influence of the fueling behavior of PHEV users is further highlighted by the fact that 2050 emissions arrive at 212 Mt in the case of SSP1/UF = 0.9 and at 370 Mt in the case of SSP1/UF = 0.7, a significant difference of 43% (Figure 4a). Conversely, the smallest influence can be observed in SSP5 with a 9% difference in 2050 emissions (Figure 4c).

### 3.4 Sensitivity Analysis

**Figure 5:**
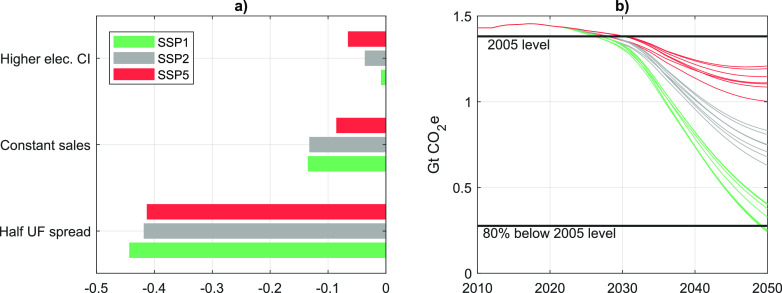
Sensitivity of the differences in cumulative well-to-wheel greenhouse gas emissions between SSP cases with low and high UF (a). The range of scenarios and sensitivity cases analyzed in this work (b). CI = carbon intensity; UF = utility factor; SSP = shared socioeconomic pathway.

Future fueling and charging behaviors are highly uncertain, and their contribution to future climate impacts will depend on the uptake of different powertrain technologies, infrastructural development, and the carbon intensity of energy sources. In the last section, we explored three plausible pathways of the US passenger vehicle market and the energy supply sector and quantified their GHG and energy use implications. In this section, we further alter some key variables to test the robustness of our results. Figure 5a shows the influence of three key variables on the relative difference in cumulative WTW GHG emissions between the cases with high and low UF. This difference is the area highlighted in gray in Figures 4a–c.

Little surprisingly, by far the largest influence is due to the spread between high and low assumed UF. Lowering this spread by half reduces the difference in results by almost the same amount (41–44%). As can be seen in Figure 3, LAVE-Trans assumes a substantial increase in future vehicle sales. Holding annual vehicle sales steady at the 2020 level of roughly 16 million units per year implies a cumulative reduction of total vehicle sales over the 2020–2050 period by 14%–20%, depending on the scenario. Accordingly, differences in cumulative emissions change by 8–13%. Keeping the carbon intensity of electricity in SSP5 at the 2020 level reduces the difference in cumulative emissions by about 7%. The influence of these parameters is also reflected in the various diverging emissions pathways as shown in Figure 5b. Only three cases reach the 80% reduction in GHG emissions relative to 2005 hypothesized by several authors.^24,39,40^ All of these cases require socioeconomic development in line with SSP1 and favorable PHEV fueling behavior (UF = 0.9). The base case (SSP1/UF = 0.9) reaches an 84.7% reduction. From there, lower vehicle sales reduce emissions further but only by 0.5 percentage points, while a slightly higher carbon intensity of electricity (59 vs 50 g CO2e/kWh) would offset emission reductions by 0.6 percentage points. We caution the reader that further significant variation could be introduced by changing the vehicle choice parameters in LAVE-Trans,^28^ which is not something we have done in this work.

## 4 Discussion

### 4.1 Implications for the US Carbon Budget

In this work, we find that the fueling behavior of PHEV drivers can determine the discharge of up to 1.9 Gt CO_2_e over the next 30 years. These results are not insignificant when one considers that US fuel economy standards led to a cumulative reduction of about 17 Gt CO_2_e of combustion emissions over the last 43 years.^2^

Further, implications for emission reduction targets and the carbon budget are substantial as well. Estimates of the US carbon budget can range from about 80–150 Gt CO_2_ (see section S1.4). In the scenarios depicted here, WTW emissions of the LDV fleet consume between 43–55 Gt, which is about one-third to one-half of the available budget. (These estimates also include upstream emissions. Excluding these reduces cumulative emissions to about 41–51 Gt CO_2_.) This stresses the fact that it will be an immense effort to stay within the US carbon budget, and even high shares of BEVs/PHEVs and renewable electricity generation are not enough to meet ambitious climate targets if not accompanied by other measures. Achieving favorable fueling behavior of alternative vehicle drivers can be one such measure. Below we discuss some key factors that could encourage PHEV drivers to electrify the majority of their driving.

### 4.2 Nudging Consumers

#### 4.2.1 Electric Charger Availability

In a 2008 survey of nearly 2,400 households in the US, more than half of the respondents stated that they already had the ability to charge a PHEV at home (within 7.5 m/25 feet of the vehicle) but had little opportunity to charge at work or other locations.^41^ The availability of EV chargers not only at home but also at work, along highways, and in commercial areas has been found to significantly increase UFs. For example, Davies and Kurani find that a PHEV with a (24-km) 15-mile electric range achieves a UF of 30% in the absence of work charging and 50% when work charging is available.^42^ Axsen et al. find that the availability of work charging increases the UF of a PHEV with a 32-km (20-mile) electric range from 45% to 55% and similarly for a PHEV with a 64-km (40-mile) electric range from 70% to 79%.^15^ Heywood et al. cite a report prepared by EPRI which concludes that the UF of PHEVs with 16- and 64-km (10- and 40-mile) ranges varies between 27–50% and 65–80% depending on whether charging is available only at home or whether it is also available at work and commercial locations.^40^

Arguably, work chargers are even more critical for PHEV commuters than for BEV commuters. Due to the short electric range of PHEVs, it is crucial that they can be recharged at work before commuting home. In a 2013 survey by Tal et al., 70% of Prius (18-km/11-mile electric range) drivers indicated they required work charging, and so did 33% of Volt drivers (56–85-km/35–38-mile electric range); but only 5% of Leaf (BEV with 117–121-km/73–75-mile electric range) drivers required work charging.^43^ Wu et al. confirm this finding and report that workplace charging opportunities significantly increase UFs for PHEVs with an electric range below 64 km (40 miles).^18^ Zoepf et al. also confirm that ubiquitous availability of conventional chargers can double UFs but add that fast charger availability does not lead to a significant increase in UFs.^44^ A report by the National Academy of Science further stresses that the federal government should ensure that all BEV and PHEV drivers can charge their vehicles at all public charging stations, raising the convenience of electric charging.^45^

There is a range of incentives and technologies whose impact on PHEV fueling behavior seems inadequately studied, including the effect of incentivizing home charger installation, public charger reservation services through smartphone apps, removing differences in plug-design between different charger networks, the development of wireless charging, and vehicle-to-grid or vehicle-to-home applications. The optimal amount of public chargers is another key uncertainty left for future research.

#### 4.2.2 Consumer Education

Perhaps the most important incentive for charging a PHEV is the price difference between electric charging and gasoline fueling as well as the ability of the PHEV user to obtain that knowledge. Sun et al. showed that electricity prices significantly affect the timing of charging.^16^ EPA’s online fuel economy database^4^ helps consumers compare annual energy costs of most commercially available vehicles in the US market, including PHEVs and BEVs. EV Explorer^46^ combines the data from EPA’s fuel economy database with the mapping functionality of Google Maps. As a result, users can compare their annual cost of driving a BEV, PHEV, or ICEV based on their exact home and work locations, number of days of commuting, local gasoline and electricity prices, and charger availability. In addition, several smartphone apps have been developed with hundreds of thousands of EV charging locations on file and useful information on charger availability, charging status, wait times, and wait lists. Furthermore, reducing driver aggressiveness can save up to 35% of energy use in the short term and up to 21% in the long term (Table 8.2 in Heywood et al.)^40^ and can thus increase the electric range of PHEVs and UFs. Employers could link financial incentives to economical driving of their employees or pay for fuel economy training courses.

#### 4.2.3 Battery Design

Zoepf et al. find that high charger availability has a stronger impact on UFs than larger battery capacities.^44^ Regardless, it seems advisible that PHEVs offer at least an electric range comparable to the average daily US travel distance, which is about 43 km (27 miles).^3^ Sun et al. showed that PHEV drivers tend not to charge when PHEVs’ electric range is below drivers’ daily driving distance.^16^ Doubling the electric range from 18 to 35 km (11 to 22 miles), which is near the average daily distance driven, can raise the UF of a Prius from about 0.28 to about 0.42, an increase of about 50%.^44^

### 4.3 Limitations and Future Work

In this work, we estimate the GHG emissions potential of PHEV drivers adopting more or less environmentally favorable fueling behavior. The most important limitation of this work is the fact that LAVE-Trans is not able to model these behavioral changes endogenously as part of its discrete choice module. Instead it relies on exogenously defined UFs. The aim of this work is to demonstrate an upper range of potential climate impacts of variations in PHEV fueling behavior, which is why we choose to model cases with high and low, yet plausible, and externally set, fleet-wide UFs. In a sensitivity analysis, we reduce the spread between high and low assumed UFs and note the resulting changes. Surprisingly, the results are still substantial, with GHG emission differences between high and low UFs at the gigaton-scale.

Further, so-called “composite vehicles”, simplified representations of a larger, diverse group of vehicle types, are commonly estimated in vehicle choice models. It has been shown that this practice can distort vehicle sales mix estimations considerably, which is why future work should either use a broader range of vehicle options or use correction methods as described in Yip et al.^47^

Finally, since LAVE-Trans is not a full-scale energy model, additional variables had to be defined externally, such as the total amount of annual vehicle sales, carbon taxes, electricity emissions, and battery pack prices. In order to reduce the bias in our work, we provide different scenarios with various sensitivity cases, displaying a total of 21 different GHG emission outcomes (Figure 5b).

Despite modeling limitations present, our results demonstrate that fueling behavior of PHEV owners can have significant impact on fleet-wide emissions and can therefore be decisive for reaching climate targets. Future research may be directed at improving the empirical basis of our work regarding the factors that could influence fleet-wide UFs of future PHEV fleets. Further work may address the importance of fueling behavior of bifuel vehicles in other regional markets or at global scale. Integrated models should pay more attention to charging and fueling behavior and may revise implicit or potentially oversimplified assumptions.

## Supporting Information Available

Additional methodological considerations and results (PDF)
Data inputs and most important model outputs (XLSX)

## Notes

Additional research data on LAVE-Trans for this article may be accessed at https://www.nap.edu/download/18264.
