# Supplementary material for: Potential Climate Impact Variations Due to Fueling Behavior of Plug-in Hybrid Vehicle Owners in the US
Source: Environ Sci Technol. 2020 Dec 16;55(1):65–72. doi: 10.1021/acs.est.0c03796 (PMC8277143; doi:10.1021/acs.est.0c03796)
Supplement: Supplementary file 1 — es0c03796_si_001.pdf [file es0c03796_si_001.pdf]

Supporting Information

Potential climate impact variations due to fueling behavior of plug-in hybrid vehicle owners in the US

Paul Wolfram[1*] and Edgar G. Hertwich[1,2]

[1]Center for Industrial Ecology, School of the Environment, Yale University, 195 Prospect St., New Haven, CT 06511, USA

[2]Industrial Ecology Programme, Department of Energy and Process Engineering, Norwegian University of Science and Technology, 7491 Trondheim, Norway

[*]E-mail: paul.wolfram@yale.edu

Content

S1 Additional methods **S2**

S1.1 Estimating future PHEV range S2

S1.2 Scenarios of future UFs S3

S1.3 Estimating future battery costs S4

S1.4 Estimating a US carbon budget S5

S2 Additional results **S6**

S2.1 Vehicle stocks S6

S2.2 Vehicle stock emission rates S7

S2.3 Fleet energy use S8

Figures

S1 Regression of PHEV range with and without REs. S2

S2 Cost reduction rates of batteries S4

S3 Cumulative CO_2 emissions of the US following its nationally determined contribution (NDC) under the Paris Agreement S5

S4 Vehicle stocks by scenario S6

S5 Average emission rates of the vehicle stock by scenario and fueling behavior . . . S7

S6 Energy use by scenario and fueling behavior S8

S1 Additional methods

S1.1 Estimating future PHEV range

The BMW i3 REx is sometimes referred to as PHEV and sometimes as BEV with range extender (RE). Figure S1 estimates the sales-weighted range of PHEVs with and without taking REs into account. The blue regression line includes sales of the i3 REx, while the grey regression line excludes the i3 REx. The blue line shows that the sales-weighted range of PHEVs varied between 40 and 56 km (25 and 35 miles) in the past with an average of about 48 km (30 miles). When considering REs (grey line), then the sales-weighted range is a bit higher and has an upward trend.

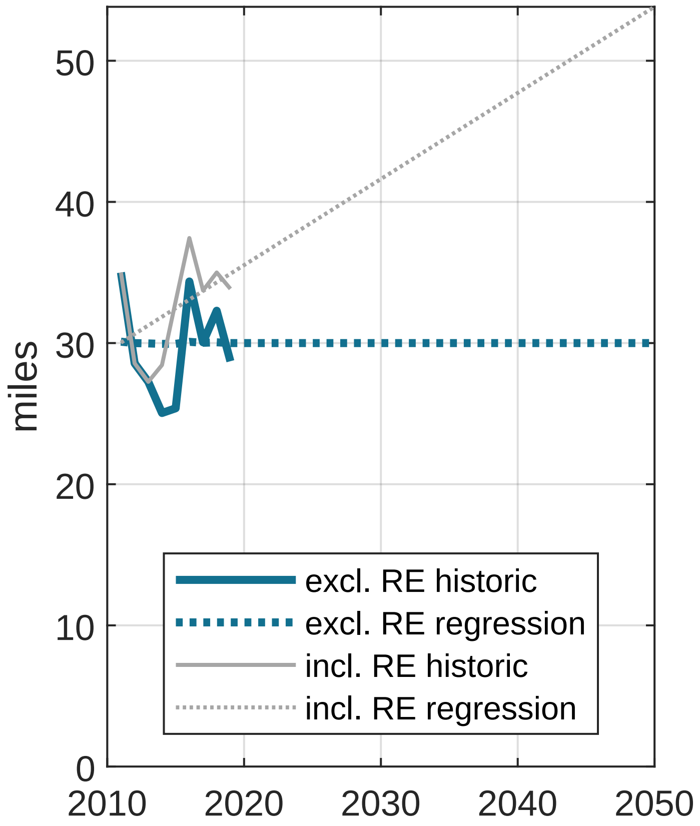

Figure S1: Regression of PHEV range with and without REs. PHEV=plug-in hybrid electric vehicle; RE=range extender.

Here we assume the constant 30-mile range (blue line) for SSP5, mainly for two reasons: Firstly, the future of the range extended version of the i3 is uncertain. Some experts believe that the REx version may be discontinued due to recent advancements in the electric range of the i3 BEV without RE.[1] Secondly, most PHEV models announced for 2020/2021 do not come with an increased all-electric range over previous models,[2] indicating that future PHEVs may not necessarily offer longer electric ranges compared to current ones. Conversely, we assume that PHEV range in SSP1 closely follows the grey line and grows up to about 60 miles by 2050. In SSP2, PHEV range grows up to an intermediate 45 miles (compare with Figure 1a in the main text).

S1.2 Scenarios of future UFs

In line with Plötz et al.[3], MacPherson et al.[4] and EPA data[5] we estimate that a current PHEV with a 30-mile electric range yields a UF of about 0.6, while a PHEV-45 (45-mile electric range) reaches a UF of about 0.75, whereas a PHEV-60 can reach a UF of about 0.9. However, we also assert that certain factors could significantly reduce these UFs. For example, the International Transport Forum (ITF)[6] assumes that the UF of a current average PHEV-45 can be significantly reduced by about 45% if used as a ride-sourced vehicle (Lyft, Uber). Assuming that 20% of all future travel demand could be met by ride-sourced vehicles, a fleet-average UF of 0.75 could fall down to 0.7 ($.7 = .2 \times .75 \times (1 - .45) + (1 - .2) \times .75$). Furthermore, if future PHEV and BEV sales grow at a speed at which the deployment of electric charging infrastructure cannot keep up with, PHEV drivers may choose to drive more miles on gasoline. A limited network of chargers could potentially imply that certain chargers be reserved exclusively for pure BEVs.[1] If this effect would further reduce PHEV charging by 15%, the average UF of 0.7 could further drop to about 0.6 ($.6 = .7 \times .85$). These back-of-the envelope calculations need to be backed by more empirical data in the future, but nevertheless highlight the potential variation in average UFs of the PHEV fleet. Other influential effects may include potential future incentives for favorable fueling behavior, raising the UF, or increased use of PHEVs for business travels or as government vehicles, which in turn could lower the UF.

[1] https://www.carthrottle.com/post/experts-want-plug-in-hybrids-banned-from-public-chargers/

S1.3 Estimating future battery costs

BEV batteries: As laid out in the main text, BEV battery packs are assumed to fall from about 600 USD/kWh in 2010 to 50 USD/kWh by 2050 in SSP1, 100 USD/kWh in SSP2 and 150 USD/kWh in SSP5. The corresponding annual rates of cost reduction can be seen in Figure S2a and b. The initial cost reduction rate is based on Nykvist and Nilsson[7] who report that costs declined by about 14% per year between 2007 and 2014. Cost reductions rates are assumed to fall more quickly in SSP5 and more slowly in SSP1.

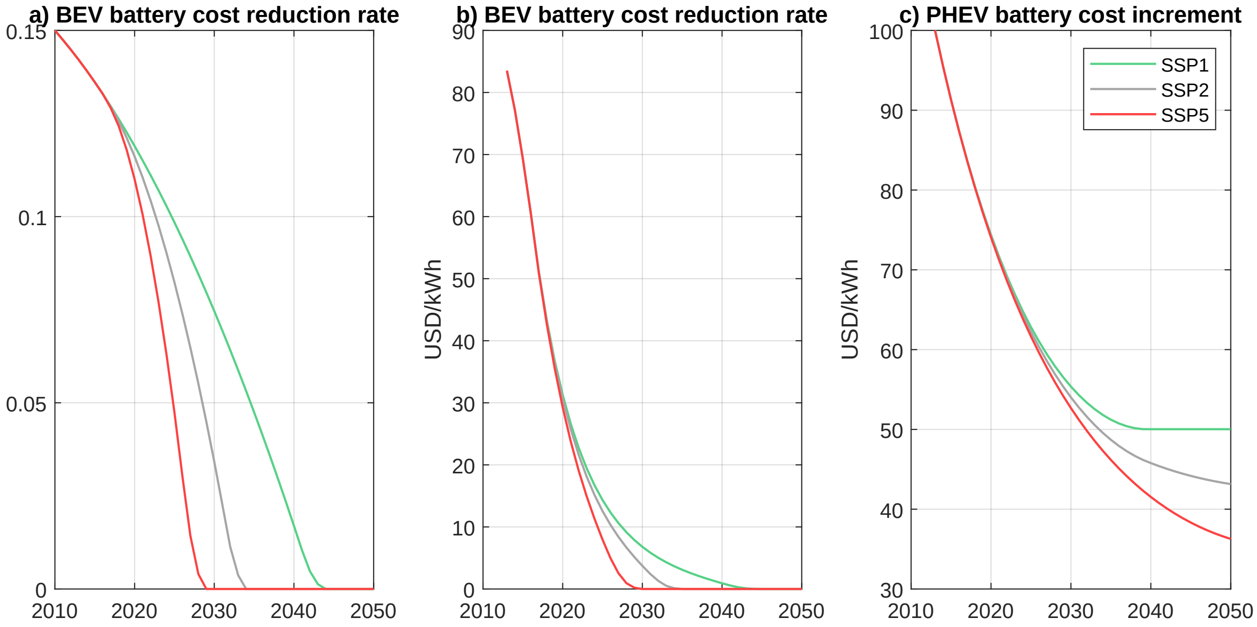

Figure S2: Cost reduction rates of batteries. Relative (a) and absolute (b) cost reduction rates of BEV battery packs. Absolute cost increment of PHEV battery packs over BEV battery packs (c). SSP=shared socio-economic pathway. PHEV=plug-in hybrid electric vehicle; BEV=battery electric vehicle.

PHEV batteries: As mentioned in the main text we assume that PHEV batteries are slightly more costly but their cost increment over BEV batteries falls over time (Figure S2c). We further assume that this cost increment falls slower in SSP1, since investments are primarily targeted at BEV batteries, and faster in SSP5. More details can be found in the accompanying spreadsheet file.

S1.4 Estimating a US carbon budget

Over the time period 2000–2050, a global carbon budget of roughly 1,000 Gt CO_2 yields about a 75% probability of staying below a global temperature increase of 2°C.[8] Assuming a grandfathering policy in which the US would be allowed to continue to emit 15% of global CO_2 emissions as has been the case historically,[2] the US would be assigned a carbon budget of 150 Gt CO_2. Assuming that annual per capita emissions were to converge globally to equal levels by 2035–2050, the US carbon budget would shrink to roughly 80–100 Gt CO_2.[9] The US has pledged to reduce annual CO_2 emissions by 26–28% by 2025 and by 80% by 2050 relative to 2005 levels. As can be seen in Figure S3, this commitment is roughly in line with the US carbon budget under grandfathering, assuming linear emission reductions.

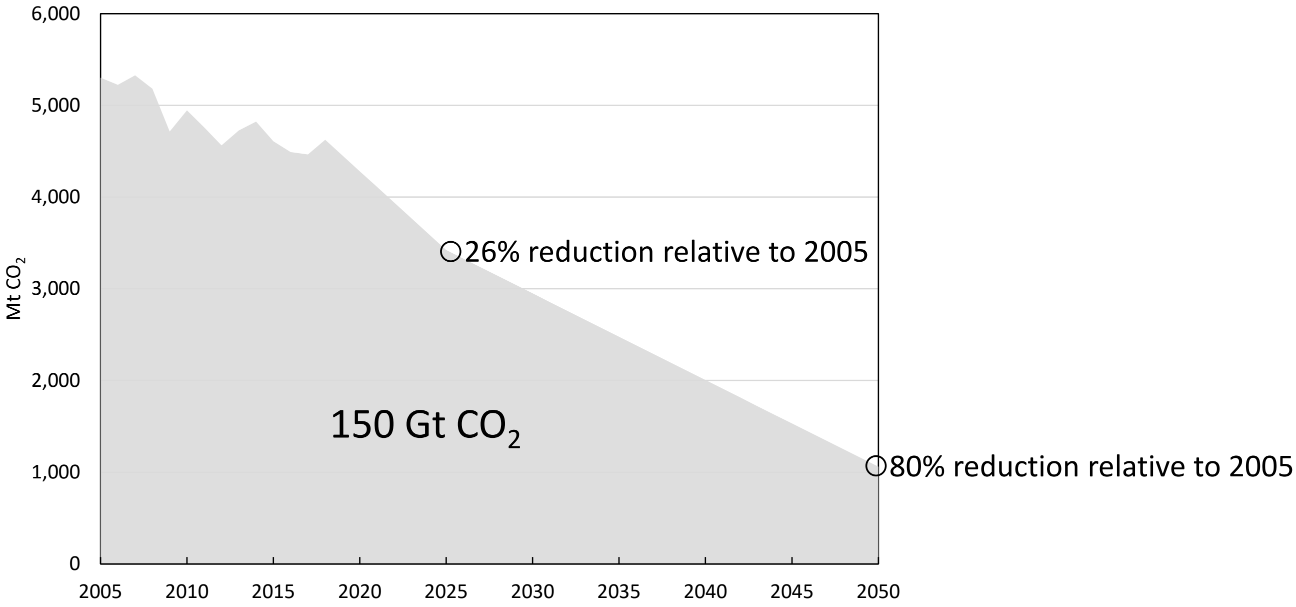

Figure S3: Cumulative CO_2 emissions of the US following its nationally determined contribution (NDC) under the Paris Agreement.

[2]https://www.globalcarbonproject.org/carbonbudget/19/highlights.htm

S2 Additional results

S2.1 Vehicle stocks

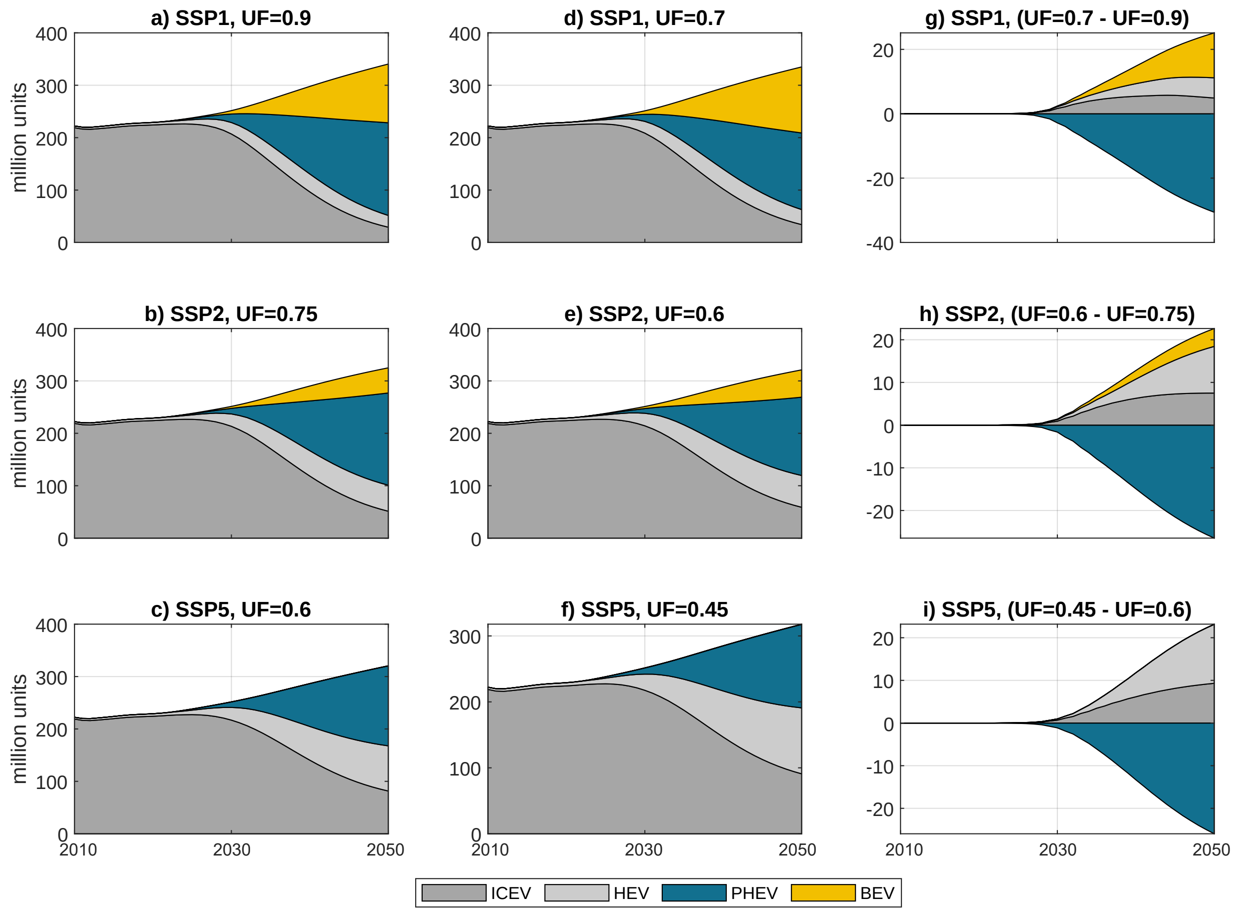

Figure S4: Vehicle stocks by scenario. High UFs (a–c). Low UFs (d–f). Differences in stocks between scenarios with low and high UFs (g–i). SSP=shared socio-economic pathway; UF=utility factor; ICEV=internal combustion engine vehicle; HEV=hybrid electric vehicle; PHEV=plug-in hybrid electric vehicle; BEV=battery electric vehicle.

S2.2 Vehicle stock emission rates

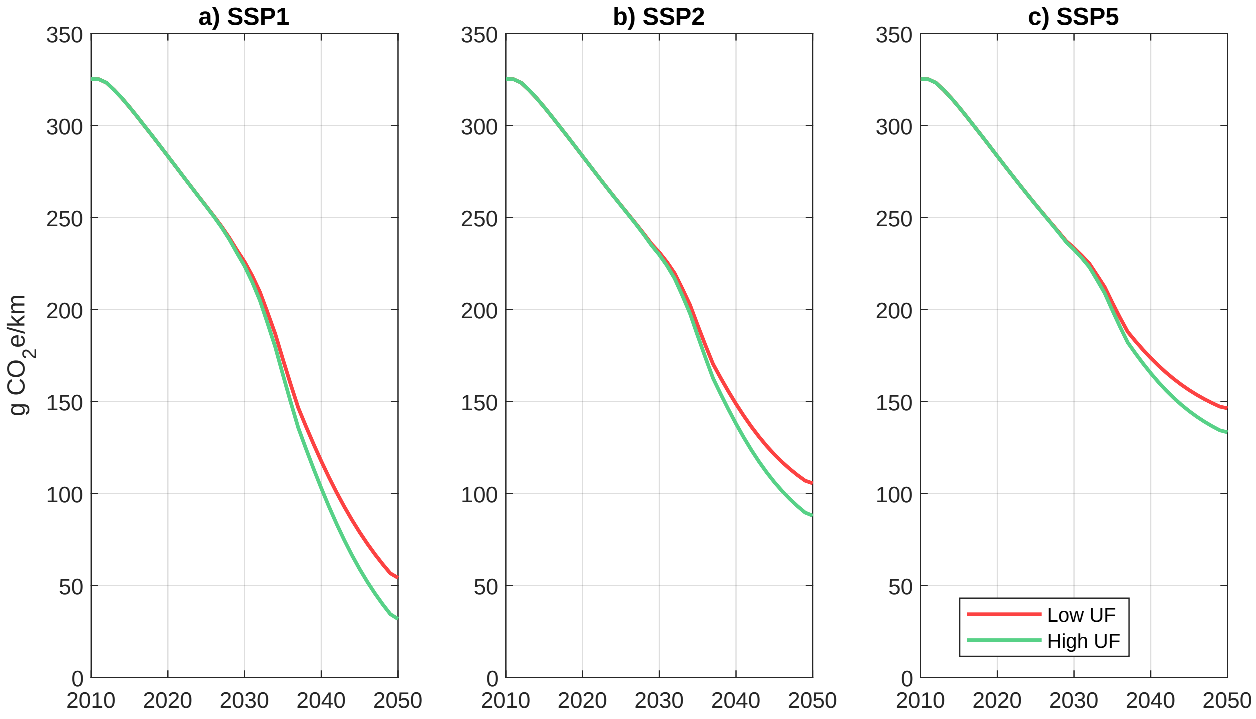

Figure S5: Average emission rates of the vehicle stock by scenario and fueling behavior. SSP=shared socio-economic pathway; UF=utility factor.

S2.3 Fleet energy use

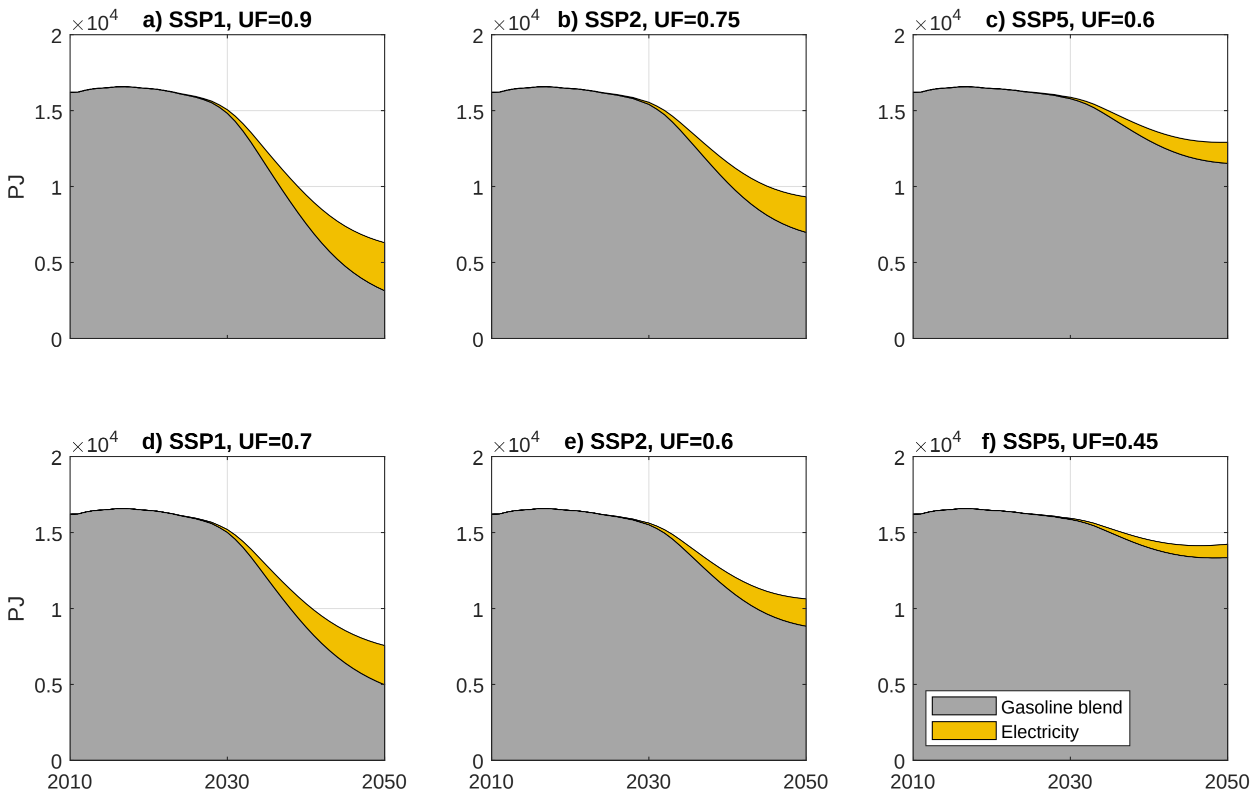

Figure S6: Energy use by scenario and fueling behavior. SSP=shared socio-economic pathway; UF=utility factor; PJ=peta joules.

References

[1] Bengt Halvorson. Batteries won: BMW confirms i3 REx range extender is on its way to extinction. Green Car Reports, 2019.

[2] Cartelligent. What new EVs and PHEVs will be released in 2020 and 2021?, 2018.

[3] P Plötz, SA Funke, P Jochem, and M Wietschel. CO_2 mitigation potential of plug-in hybrid electric vehicles larger than expected. *Scientific Reports*, 7(1):16493, 2017.

[4] Nathan D. MacPherson, Gregory A. Keoleian, and Jarod C. Kelly. Fuel economy and greenhouse gas emissions labeling for plug-in hybrid vehicles from a life cycle perspective. *Journal of Industrial Ecology*, 16(5):761–773, sep 2012.

[5] U.S. EPA. Trends Report - Light-Duty Automotive Technology, Carbon Dioxide Emissions, and Fuel Economy Trends: 1975 Through 2017, 2018.

[6] ITF. Good to go? assessing the environmental performance of new mobility. International Transport Forum (ITF), 2020.

[7] Björn Nykvist and Mans Nilsson. Rapidly falling costs of battery packs for electric vehicles. *Nature Climate Change*, 5(4):329–332, 2015.

[8] Malte Meinshausen, Nicolai Meinshausen, William Hare, Sarah C. B. Raper, Katja Frieler, Reto Knutti, David J. Frame, and Myles R. Allen. Greenhouse-gas emission targets for limiting global warming to 2C. *Nature*, 458(7242):1158–1162, April 2009.

[9] Renaud Gignac and H Damon Matthews. Allocating a 2°C cumulative carbon budget to countries. *Environmental Research Letters*, 10(7):075004, Jul 2015.
